# NODDI-derived measures of microstructural integrity in medial temporal lobe white matter pathways are associated with Alzheimer’s disease pathology and cognition

**DOI:** 10.1162/IMAG.a.950

**Published:** 2025-10-23

**Authors:** Dana M. Parker, Jenna N. Adams, Soyun Kim, Liv McMillan, Michael A. Yassa

**Affiliations:** Department of Neurobiology and Behavior and Center for the Neurobiology of Learning and Memory, University of California, Irvine, CA, United States

**Keywords:** diffusion tensor imaging, neurite orientation dispersion and density imaging, Alzheimer’s disease, white matter, amyloid-beta, tau, memory, aging

## Abstract

Diffusion tensor imaging has traditionally been used to assess white matter (WM) integrity in Alzheimer’s disease (AD). However, the tensor model is limited in modeling complex WM structure. Neurite Orientation Dispersion and Density Imaging (NODDI), a cutting-edge technique applied to multishell diffusion MRI, can offer more precise insights into microstructural features of WM integrity. We assessed whether NODDI more sensitively detects AD-related changes in medial temporal lobe (MTL) WM than traditional tensor metrics. In total, 199 older adults with multishell diffusion MRI from ADNI3 (mean age = 75 years; 60% female; cognitively unimpaired, n = 121; cognitively impaired MCI/dementia, n = 77) were analyzed. Tensor metrics of fractional anisotropy (FA) and mean diffusivity (MD), as well as NODDI metrics of Neurite Density Index (NDI) and orientation dispersion Index (ODI), were calculated for MTL WM tracts (JHU Atlas: hippocampal cingulum, fornix column/body, fornix/stria terminalis, and uncinate). A subset of participants received 18F-florbetapir or 18F-florbetaben to measure Aβ (n = 146; converted to Centiloids), 18F-flortaucipir to measure tau (n = 135), and neuropsychological testing including the Clinical Dementia Rating Sum of Boxes (CDR-SB) and memory composite score (ADNI-MEM). NODDI measures in MTL tracts were more strongly correlated with cognitive performance and AD pathology than standard tensor measures. For example, entorhinal tau was strongly associated with NDI in the cingulum hippocampus and the uncinate, and with ODI in the fornix ST. Both ODI and NDI across the majority of tracts were associated with CDR-SB and ADNI-MEM. In contrast, FA in any MTL tract was not significantly correlated with either tau or global amyloid-beta, while MD in MTL tracts showed limited correlations with pathology or cognition. NDI partially mediated the relationship between AD pathology (entorhinal tau, meta temporal tau, or Aβ) and the memory composite score. Random forest modeling showed that a combination of NODDI metrics and DTI had the strongest estimates of memory performance and clinical impairment. NODDI metrics offer more sensitive insights about MTL WM integrity in AD that could have been previously missed due to the limitations of DTI analyses. Additionally, combining NODDI with DTI yielded the strongest predictive performance for memory and clinical impairment, suggesting that the two approaches are complementary. The use of advanced diffusion acquisitions such as multishell which allows for analyses such as NODDI is crucial for the future development of disease identification and structural understanding.

## 1 Introduction

Alzheimer’s disease (AD) poses a significant public health challenge, which is exacerbated by the aging population. Currently it is estimated that roughly 6.7 million Americans are suffering from AD dementia with the potential of that number growing to 14 million in 2060 (Rajan et al., 2021). Emerging evidence indicates that development of pathology and neurobiological alterations in brain circuits occurs long before the manifestation of symptoms, possibly even decades in advance (Barthélemy et al., 2020; Braak et al., 2011; Jack et al., 2010). Consequently, there is a pressing need to establish neuroimaging biomarkers with the ability to detect early neurobiological changes that contribute to the onset of cognitive deficits in AD.

White matter (WM) tracts—large bundles of axons—enable communication between gray matter regions. The integrity of these tracts is intriguing biomarkers in AD due to their vulnerability to Alzheimer’s pathology and subsequent impact on cognitive decline. The integrity of WM within the medial temporal lobe (MTL) may have particular relevance to detecting the earliest stages of AD. The MTL, encompassing the hippocampus, amygdala, and parahippocampal gyrus, plays a vital role in spatial and episodic memory (Cutsuridis & Yoshida, 2017; Eichenbaum et al., 2007). Notably, the MTL is recognized as one of the earliest sites of atrophy (de Flores et al., 2022) and tau tangle pathology (Braak & Braak, 1995). Previous research has demonstrated strong relationships between gray matter integrity of the MTL, such as hippocampal volume (Anderson et al., 2012; Xiao et al., 2023) and disease progression. Further, MTL regions have been shown to have declining WM microstructure associated with normal aging (Teipel et al., 2014). Biomarkers of MTL WM integrity in the earliest stages of AD have been less studied (Alm & Bakker, 2019; Magalhães et al., 2023), though they may provide more sensitive information on structural decline.

**Fig. 1.**
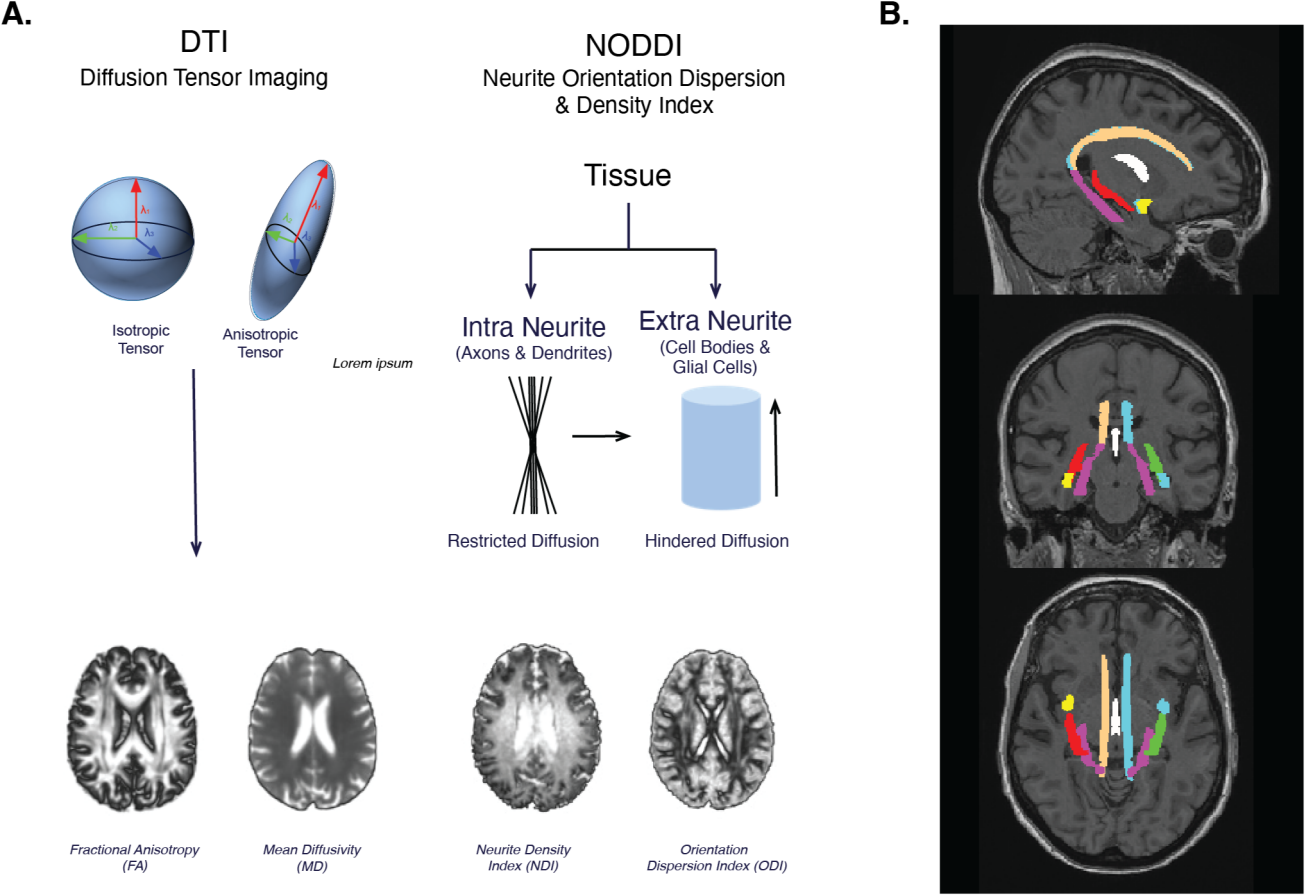
(A) Overview of Diffusion Tensor imaging and overview of NODDI. (B) Example of JHU WM Atlas ROIs.

Diffusion magnetic resonance imaging (dMRI), a noninvasive neuroimaging method, holds great promise in assessing the integrity of WM pathways. By employing dMRI, subtle and early changes in the brain’s WM can be detected (Márquez & Yassa, 2019; Teipel et al., 2014). Diffusion Tensor Imaging (DTI) can be used to calculate scalar indices derived from eigenvalues which are employed to describe anisotropy (Teipel et al., 2014). The most frequently utilized DTI indices include mean diffusivity (MD), which reflects the overall mean squared displacement of water molecules, and fractional anisotropy (FA), a measure of the magnitude of molecule diffusion that can be equated to anisotropic (i.e. directional) diffusion (Fig. 1A) (Le Bihan, 1995; Teipel et al., 2014). Both FA and MD are widely used as proxies for observing microstructural tissue changes during brain aging (Ciccarelli et al., 2008). However, this tensor model poses major limitations as it fails to capture the complex architecture of WM characterized by crossing, bending, twisting, and kissing fibers (Jensen et al., 2005).

An alternative approach in dMRI involves tensor-free modeling, which encompasses various methods such as Q-shell imaging, Diffusion Kurtosis Imaging, and freewater elimination modeling (Jensen et al., 2005; Pasternak et al., 2009). Neurite Orientation Dispersion and Density Imaging (NODDI), another emerging tensor-free method, has the distinct advantage of allowing the examination of multiple compartments through multishell imaging, thereby offering a closer representation of the brain’s actual architecture (Motovylyak et al., 2022; Timmers et al., 2016; Zhang et al., 2012). Specifically, NODDI incorporates three microstructural compartments: intra-cellular, extracellular, and cerebrospinal fluid (CSF) (Zhang et al., 2012). NODDI generates metrics such as Neurite Density Index (NDI), which represents the portion of the tissue that is composed of axons and dendrites, and Orientation Dispersion Index (ODI), which reflects the angle variation and configuration of the neurite structure (Fig. 1A) (Billiet et al., 2015). Previous research has validated NODDI measures against histological markers, revealing robust associations with compartment-specific indicators of neurite integrity. For example, Colgan et al. (2016) reported that NODDI, in a mouse model of human tauopathy (rTg4510), demonstrated the capacity to establish correlations within brain regions affected by tau. Remarkably, NDI outperformed the conventional FA metric by being the sole metric capable of detecting a correlation with tau burden in the hippocampus (Colgan et al., 2016).

Prior studies have begun to demonstrate associations between NODDI metrics and tau deposition or cognition in Alzheimer’s disease, including voxel-wise associations in both gray and white matter regions (Sone et al., 2020; Tian et al., 2023; Wen et al., 2019; Weston et al., 2023). However, few studies have directly compared tensor-based and NODDI models within the same sample, across multiple medial temporal white matter pathways, and within a multisite harmonized dataset. The present study extends this literature by evaluating both tensor and NODDI metrics in parallel, applying mediation models to test mechanistic pathways linking pathology and cognition, and using machine learning approaches to compare estimation performance across models.

A growing number of studies have applied NODDI to examine microstructural alterations in Alzheimer’s disease and have reported that NODDI metrics may outperform conventional DTI measures in detecting early pathological changes (Gallagher et al., 2023; Moody et al., 2022; Vogt et al., 2020, 2022; Yu et al., 2024). Building upon this prior work, the present study leverages harmonized multishell diffusion data from the ADNI3 cohort, applies NODDI and tensor models across multiple medial temporal white matter pathways, incorporates mediation analyses linking pathology and cognition, and evaluates classification performance using machine learning approaches.

While NODDI represents a promising improvement to dMRI analyses of WM integrity, it has not yet been thoroughly investigated in the context of AD specifically within the ADNI cohort, since prior to ADNI3 there was no multishell diffusion-weighted imaging (DWI) sequence. The objective of this study is to investigate whether NDI and ODI metrics, obtained through NODDI models of dMRI, exhibit improved performance of detecting AD pathology in MTL WM. Given that memory loss is a hallmark of early Alzheimer’s disease, we focused on memory outcomes and pathology in relation to early alterations in major white matter pathways within the medial temporal lobe that are particularly vulnerable in the initial stages of the disease, specifically the cingulum, fornix, and uncinate fasciculus. We hypothesize that NODDI metrics will enhance sensitivity in detecting microstructural changes in WM across our population of cognitively unimpaired, mild cognitively impaired, and individuals with AD dementia.

## 2 Methods

### 2.1 Data source

Data used in the preparation of this article were obtained from the Alzheimer’s Disease Neuroimaging Initiative (ADNI) database (adni.loni.usc.edu). The ADNI was launched in 2003 as a public–private partnership, led by Principal Investigator Michael W. Weiner, MD. The primary goal of ADNI has been to test whether serial MRI, positron emission tomography (PET), other biological markers, and clinical and neuropsychological assessment can be combined to measure the progression of mild cognitive impairment (MCI) and early AD. For up-to-date information, see www.adni-info.org. Ethical approval was obtained by the ADNI investigators at each participating ADNI site. All participants provided written informed consent.

### 2.2 Study participants

All participants from the ADNI3 protocol were evaluated for inclusion of the multishell DWI sequence giving us a total of 230 initial participants for this study. After initial quality control to assess scan completeness, protocol alignment, and scanner harmonization (ensuring all scans were acquired on Siemens MRI machines), visual inspection was performed to identify issues such as motion and susceptibility artifacts (n = 5), registration failures (n = 8), and missing b-shells (n = 2). Following this process, 199 participants were retained for analysis. Due to a low number of participants with a dementia diagnosis (N = 12), individuals diagnosed with MCI were combined with participants diagnosed with dementia for a Cognitively Impaired (CI) group (N = 77), which were compared against a Cognitively Normal (CN) group (N = 121). Table 1 provides a breakdown of participant demographics. The cognitively impaired group included individuals classified as either mild cognitive impairment (n = 65) or mild Alzheimer’s disease dementia (n = 12) per ADNI3 clinical criteria. The dementia cases were all diagnosed as probable AD. Inclusion of both MCI and mild dementia cases allowed us to capture a broader continuum of AD-related cognitive and biomarker variability for dimensional analyses. Molecular biomarker data were available for a subset of participants. Amyloid PET (florbetapir or florbetaben, converted to Centiloids) was available for 146 participants, with 25 cognitively normal (20%) and 32 cognitively impaired participants (41%) classified as amyloid positive (Centiloid > 18). Tau PET (flortaucipir) data were available for 135 participants; mean entorhinal tau SUVRs were 1.8 (CN) and 1.9 (CI), and mean meta-temporal tau SUVRs were 1.6 (CN) and 1.7 (CI). These molecular biomarkers were used in correlational and mediation models throughout the analyses.

**Table 1.** Demographic breakdown.

		CI (N = 77)		
	CN (N = 121)	MCI (N = 65)	AD (N = 12)	p-value
Age (range)	71(51-90)	72.3(49-90)	76.6(59-89)	0.09
Aβ+ (%)	25(20)	25(39)	7(59)	0.00002^†^
APOE4 (%)	28(23)	19(29)	6(50)	0.02^†^
Entorhinal tau (std)	1.8(0.45)	2.1(0.7)	3.1(0.8)	0.001
Meta temporal tau (std)	1.6(0.25)	1.8(0.6)	2.6(0.9)	0.0001
Sex: female (%)	79(65)	29(45)	7(59)	0.01^†^
Years of education (std)	17(2.36)	16(2.6)	15(2.7)	0.04
Race				0.4^†^
White (%)	94(78)	56(86)	8(67)	–
Black (%)	19(16)	5(8)	3(25)	–
Asian (%)	6(5)	2(3)	0	–
More than one (%)	2(2)	2(3)	1(8)	–
Ethnicity				0.6^†^
Hispanic/Latino (%)	2(2)	3(4)	0	–
Unknown (%)	1(0.8)	1(1)	0	–
Not Hispanic/Latino (%)	118(98)	61(94)	12(100)	–
CDR-SB (std)	0.03(2.36)	1.6(1.3)	5.4(3.2)	0.00001
ADNI-MEM (std)	1.12(0.69)	0.3(0.7)	-0.6(0.5)	0.00001

CN, cognitively normal; CI, cognitively impaired; CDR-SB, Clinical Dementia Rating Sum of Boxes; ADNI-MEM, ADNI memory composite score; std, standard deviation. p-Values are statistical comparisons between CN and CI groups.

† denotes comparison via chi-squares test; otherwise all comparisons were independent samples t-tests.

### 2.3 Structural and diffusion MRI processing

To ensure the homogeneity of the scan data across different sites, only scans obtained using Siemens MRI machines were selected for analysis. This decision aimed to minimize potential variations that could arise from using different machine manufacturers and ensure a more consistent and standardized dataset for the study. To further assess potential site-specific effects, we conducted our regression models including scanner site as a control as well as age, sex, and education. These analyses did not reveal significant site differences for any of the NODDI metrics across the regions of interest (all p > 0.1), suggesting that site variance was minimal after limiting to Siemens Prisma scanners. All participants were scanned using the ADNI 3.0 Advanced MRI scanning protocol, the first ADNI protocol to include multishell scan sequences. Each participant underwent a magnetization prepared rapid acquisition gradient echo (MPRAGE) sequence with the following parameters: echo time (TE)=2.98 ms, repetition time (TR) = 2300 ms, inversion time=900 ms, flip angle=10°, field of view (FOV)=208 × 240 × 256 mm^3^, acquired resolution =1 × 1 × 1 mm. MRI parameter specifics are given at https://adni.loni.usc.edu/data-samples/adni-data/neuroimaging/mri/mri-scanner-protocols/ T1-weighted images were corrected using Freesurfer 7 pipeline, which corrected for head motion and intensity inhomogeneity, then the removal of non-brain tissue. After structural scans were quality checked, corrected hippocampal volume was calculated using intracranial volume and raw hippocampal volume Freesurfer output.

Diffusion scans were acquired via the following parameters: 232 x 232 x 160 mm @ 2 x 2 x 2 mm; TE = 71 TR = 3300. Three separate shells were collected at the following b-values: 500, 1000, and 2000 s/mm² for a total of 112 directions. For the tensor metrics (FA and MD), the 1000 s/mm² shell was chosen as it was the b-value that was used in the single shell diffusion scan in the non-advanced ADNI3 protocol. Scans were preprocessed using MRtrix 3 (Tournier et al., 2012) (www.mrtrix.org). Diffusion data preprocessing included denoising using MRtrix3’s dwidenoise function, correction for Gibbs ringing artifacts (Kellner et al., 2016), susceptibility-induced distortions using fieldmap-based EPI distortion correction, eddy current and motion correction using FSL’s eddy tool, and bias field correction. These steps were all completed prior to manual quality control. Tensor metrics were extracted via MRtrix3 and maps were created for FA and MD (Fig. 1A). The Microstructure Diffusion Toolbox (MDT) (Harms et al., 2017) was used to calculate NDI and ODI maps (Fig. 1A). Although FISO may provide useful insights into extracellular free water, it is particularly susceptible to partial volume effects from CSF, especially in small MTL tracts such as the fornix. Given our focus on robust tract-level estimates of tissue microstructure, we prioritized NDI and ODI as the most biologically interpretable and reliable NODDI metrics for this dataset.

The following MTL ROIs from the JHU DTI-based WM atlas (https://identifiers.org/neurovault.collection:264) were used in this analysis (Fig. 1B): cingulum cortex, hippocampal cingulum, fornix column body, fornix ST (stria terminalis), and uncinate. Following preprocessing of the dMRI, images metrics for both NODDI and DTI were transformed to MNI space, and then the mean values of FA, MD, NDI, and ODI were extracted from each tract. Given the small size and partial volume susceptibility of certain medial temporal tracts, particularly the fornix ST, we implemented multiple safeguards. All diffusion images were visually inspected after preprocessing and spatial normalization to verify accurate registration and anatomical localization of the fornix ST across participants. ROI extraction was performed using standardized atlas-based masks in MNI space to ensure consistency. Importantly, the NODDI model itself provides partial mitigation of partial volume effects by explicitly estimating intracellular, extracellular, and CSF compartments, thereby reducing contamination from surrounding tissues.

### 2.4 Clinical and cognitive measures

We analyzed the Clinical Dementia Rating Scale—Sum of Boxes score (CDR-SB), the Clinical Dementia Rating Scale—Memory (CDR-MEM) and ADNI-MEM as primary measures. The CDR-SB is a widely used clinical measure that assesses the severity of dementia in patients at a given time point. The “sum of boxes’’ component of the CDR provides enhanced precision for tracking changes over time and minimizes potential computational errors. The memory component of the CDR scale provides additional insights into the population based on their overall memory performance. Out of 199 included participants, 195 successfully completed the CDR and thus were included in the analyses of this measure (Morris, 1993).

To assess memory performance, we used the ADNI-MEM, a composite memory score derived from the cognitive battery as described by Crane et al. (2012). We opted to utilize this measurement as it provides a combined single memory score that serves as a robust and comprehensive representation of memory performance (Crane et al., 2012). Out of the 199 participants, 186 had data available for ADNI-MEM.

### 2.5 Positron emission tomography

PET imaging protocols included 18F-flortaucipir (FTP) to measure tau pathology and either 18F-florbetapir (FBP) or 18F-florbetaben (FBB) to measure Aβ pathology (depending on availability at time of collection). All preprocessing was completed by the ADNI PET core at UC Berkeley. Full details on PET processing and acquisition are available at the ADNI website https://adni.loni.usc.edu/wp-content/uploads/2012/10/ADNI3_PET-Tech-Manual_V2.0_20161206.pdf.

FTP data were analyzed between 80 and 100 min post-injection across four 5-min frames, normalized using an inferior cerebellum gray reference region, and a Rousset approach for partial volume correction (Baker et al., 2017; Rousset et al., 1998). The mean standardized uptake value ratio (SUVR) values of FreeSurfer regions (version 7.1.1) were used. We focused on the mean SUVR of the entorhinal cortex and a meta-temporal ROI, which is a composite of the following regions: entorhinal, parahippocampal cortex, amygdala, fusiform, medial temporal, and inferior temporal (Jack et al., 2010).

FBP data were analyzed between 50 and 70 min post-injection, while FBB data were analyzed between 90 and 110 min post-injection. both across four 5-min frames. Both FBP and FBB were normalized using a whole cerebellum reference region. Global amyloid-beta was calculated using a cortical summary region consisting of Freesurfer-defined (version 7.1.1 processing) frontal, anterior/posterior cingulate, lateral parietal, and lateral temporal regions (Landau et al., 2012, 2013). To enable combination of the two tracers, regional and global FBP and FBB values were converted to the Centiloid scale, and a threshold of 18 Centiloids was used on the cortical summary region to determine amyloid-beta positivity (Royse et al., 2021).

### 2.6 Statistical analysis

All statistical analyses were performed in Jamovi 2.3 (The Jamovi Project 2022) or Python (version 3.8). Mediation analyses were performed in Jamovi using the medmod module. Correlation analyses performed were partial correlations controlling for age, sex, and education (Kim, 2015). All p-values were corrected using the Bonferroni–Holm method in R (version 3.6.2) https://rdocumentation.org/packages/stats/versions/3.6.2 (correcting for five comparisons) using the p.adjust function from the “stats” package in R. We also conducted exploratory subgroup analyses, repeating partial correlations separately within CN and CI groups to evaluate whether findings were driven by diagnostic group. See Supplementary Tables 6A-B, 7A-B, 8A-B, and 9A-B.

Random forest classification models were used to estimate CDR-SB classification (CDR > 0 vs CDR=0) and ADNI-MEM (mean split). Models were constructed as (1) tensor only, (2) NODDI only, and (3) combined models (best tensor, NODDI, and demographics). Random forest (Breiman, 2001) is a type of ensemble machine learning algorithm that is widely used for classification or regression models including multidimensional variables (Delgado et al., 2014). We developed and implemented RF algorithms in Python using the Scikit-Learn library (Pedregosa et al., 2011). In total, 100 decision trees consisting of different combinations of estimation variables were built for each model. Model performance was evaluated using area under the curve (AUC) of the receiver operating characteristic (ROC) curves, which was plotted from all the estimated values of the test dataset using leave-one-out cross validation (LOOCV). The LOOCV design was specifically chosen given the small sample size (Falahati et al., 2014) and built n classification models using n-1 subject each time which then utilized the classifier to determine the class of the left-out subject. Sensitivity and specificity were computed for each classification. We also performed bootstrapping (1000 simulations) to estimate 95% confidence intervals for the AUCs. Feature importance was also obtained using the Gini impurity index (Breiman, 2001).

## 3 Results

### 3.1 NODDI measures show stronger associations with AD-related outcomes

We aimed to determine whether using NODDI metrics could more sensitively account for complexities in WM tract microstructure, thus revealing stronger associations with outcome measures. Our two primary NODDI outcome measures were NDI, reflecting the density of how the neurites are packed, and ODI, reflecting orientation and directionality of the neurites (Fig. 1A).

**Fig. 2.**
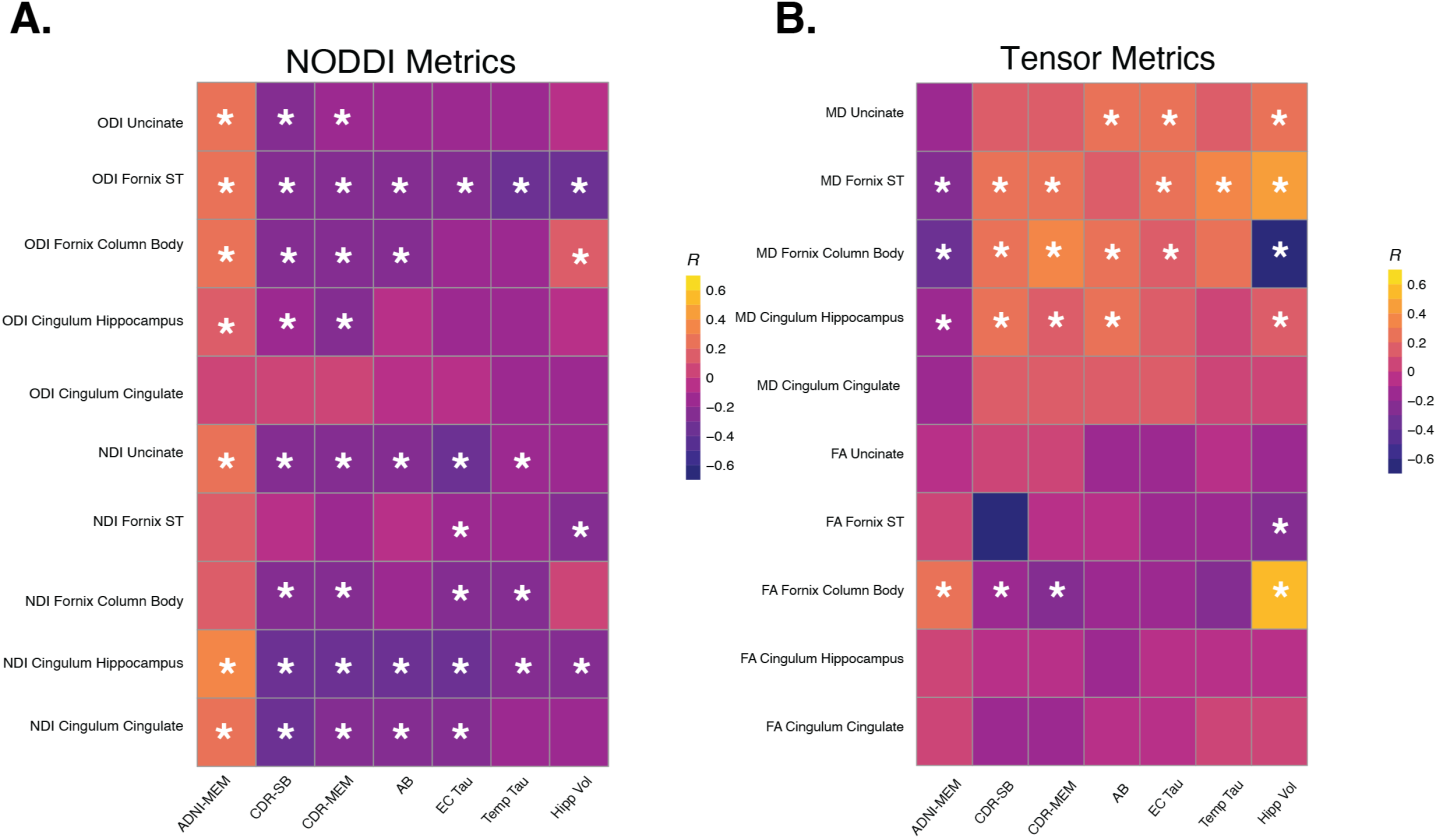
Correlation matrices between NODDI metrics (A) and tensor metrics (B) showing relationships between outcome measures and WM integrity of each ROI. NDI, neurite orientation index; ODI, * indicates p-value ≤ 0.05 after correction. Abbreviations: Fornix ST = fornix stria terminalis, CDR-SB = Clinical Dementia Rating Scale Sum of Boxes, β-amyloid = amyloid beta, EC Tau = entorhinal tau, Temp Tau = meta temporal Tau, Hipp Vol = hippocampal volume.

We first tested relationships between NODDI metrics of the MTL with clinical and cognitive performance, specifically the CDR-SB, CDR-MEM, and ADNI-MEM. Partial correlation analysis of NODDI results revealed that NDI consistently exhibited a larger frequency of significant correlations with outcome measures when compared with ODI across all outcome measures, as shown in Figure 2A and Supplementary Figure 1. Specifically, NDI in the cingulum cortex and hippocampal cingulum regions demonstrated consistent and significant correlations with cognitive outcomes (ps_FDR_ < 0.05). In contrast, ODI only showed significant associations predominantly in the fornix and uncinate regions. Interestingly, ODI displayed consistent correlations across all three outcome measures (CDR-SB, ADNI-MEM, and hippocampal volume) in the fornix ST (ps_FDR_ < 0.001). In most tracts, NODDI metrics showed significantly stronger associations with cognitive performance compared with FA; however, in the cingulum cortex, the difference between ODI and FA was not statistically significant. To determine whether NDI indeed had a stronger association than ODI, we formally tested the strength of the associations using a Steiger’s z test [41]. We found that the correlation between NDI of the hippocampal cingulum regions was stronger than ODI hippocampal cingulum regions (z = 2.0251, p-value = 0.0214). This was also the case for NDI cingulum cortex and ODI cingulum cortex (z = 2.0560, p-value = 0.0199). Additional Steiger Z-tests indicated that ODI outperformed NDI in estimating memory performance in the fornix ST (z = 2.5486, p = 0.0054) but not the uncinate fasciculus (z = -0.35, p = 0.64). Across the full set of medial temporal tracts, NDI and ODI demonstrated region-specific strengths, suggesting complementary sensitivity across white matter pathways relevant to memory. These results demonstrate that among the NODDI metrics, a significant decrease in neurite density is correlated with a decline in cognitive performance. While ODI showed fewer relationships, it was still able to show that reduced neurite orientation was associated with worse cognitive performance. We next tested associations between NODDI with Alzheimer’s pathology, specifically global Aβ, and both entorhinal and meta-temporal tau. Both NDI and ODI of various WM tracts exhibited significant correlations with pathology, with entorhinal tau demonstrating the greatest number of associations across both NODDI metrics. Entorhinal tau was significantly associated with NDI in the hippocampal cingulum (r = -0.375; p < 0.001) and in the uncinate (r = -0.373; p < 0.001), and with ODI in the fornix ST (r = -0.288; p = 0.004). Notably, ODI of the fornix ST showed significant associations with both tau ROIs (entorhinal r = -0.288; p < 0.001; meta-temporal r = -0.319; p < 0.001). Z tests showed that neither the NDI hippocampus nor the uncinate was more significant than ODI fornix ST. Our analyses showed that with greater tau pathology, there was reduced neurite density as well as a reduction in the orientation of the neurites, demonstrating a reduction in diffusion properties in regions impacted by AD-related tau pathology.

Finally, we investigated associations between NODDI measures and hippocampal volume as a proxy of neurodegenerative processes. Notably, hippocampal volume exhibited relatively fewer significant associations with the NODDI metrics. Hippocampal volume was only significantly associated with ODI of the fornix ST (r = -0.301; p < 0.001) and NDI of the hippocampal cingulum (r = -0.226; p = 0.0125) and fornix ST (r = -0.215; p = 0.01). Exploratory subgroup analyses stratifying by cognitive status demonstrated largely consistent patterns of association across both groups, with attenuated effect sizes observed in the CN group due to reduced range of pathology and cognitive impairment. Full subgroup correlation results are provided in Supplementary Tables 4A, 4B, 5A and 5B.

### 3.2. FA shows fewer relationships between cognitive and pathological outcome measures

We then examined the relationship between standard tensor-based diffusion metrics, namely FA and MD, with these same cognitive and pathology outcome measures (Fig. 2B). Among the three cognitive outcome measures, there were no significant correlations with FA of any WM tracts assessed. However, MD of the hippocampal cingulum (r = -0.185; p = 0.02), fornix ST (r = -0.273; p<0.01), and uncinate (r = 0.244; p = 0.01) demonstrated strong associations with ADNI-MEM. CDR-SB and CDR-MEM also showed results similar to ADNI-MEM (Fig. 2B; Supplementary Table 2A). No other regions demonstrated significant findings in relation to MD.

We next assessed relationships between tensor metrics with AD pathology and hippocampal volume. There were no significant correlations between FA in any tract with either global Aβ or tau pathology in entorhinal cortex or meta-temporal ROI (ps_FDR_ > 0.075). However, FA of the fornix ST and fornix column body had significant relationships with hippocampal volume. MD across multiple tracts such as the fornix ST and fornix column body did show relationships with pathology and hippocampal volume, as indicated in Figure 2B and Supplementary Table 2A.

Using the Steiger Z test, we found that NDI was significantly more strongly correlated with cognitive outcomes than FA (z > 2.7; p < 0.012). We also found that ODI was significantly more strongly correlated with cognitive outcomes than FA, except in the cingulum cortex. Correlations between outcome measures and NDI were significantly stronger than with MD, further confirming our hypothesis that NDI can be considered a more sensitive metric for AD-related white matter changes.

Together, our results show that worse cognitive and pathological outcomes are associated with significant decreases in NDI and ODI which reflects a loss of neurite density as well as an increase in neurite dispersion. This demonstrates the high sensitivity of NODDI metrics to detect these processes. In contrast, for the tensor metrics, increases in MD, but not FA, were associated with these measures, which highlights the reduced sensitivity of traditional DTI models, especially the commonly used metric of FA.

### 3.3 Mediation models show strength of NODDI in showing mechanistic underpinnings of neurodegeneration

**Fig. 3.**
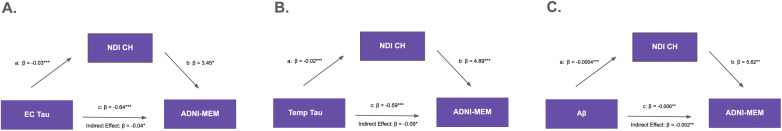
Mediation models demonstrating the relationship of NODDI’s NDI metric on AD pathology. All correlations shown here are partial. (A) NDI mediates the relationship of entorhinal tau and ADNI-MEM .(B) NDI mediates the relationship of meta temporal tau and ADNI-MEM. (C) NDI mediates the relationship between β-amyloid and ADNI-MEM. NDI, neurite orientation index; ODI, * indicates p-value ≤ 0.05, ** indicates p-value ≤ 0.001, *** indicates p-value ≤ 0.0001. Abbreviations: NDI = Neurite Density Index, CH = hippocampal cingulum, EC Tau = entorhinal tau, Temp Tau = meta temporal tau, β-amyloid = amyloid beta.

We next tested potential mechanistic links between pathology, NODDI metrics of WM integrity, and cognitive performance. We chose the hippocampal cingulum due to its strong associations found in our correlation models and known role in memory processing. We chose to examine NDI rather than ODI from NODDI also due to its more sensitive performance in our statistical models. Additionally, we did not find relationships with MD or FA and either entorhinal or meta temporal tau, so we were unable to use these metrics as part of our mediation modeling. ADNI-MEM was selected as the cognitive outcome measure due to previous work demonstrating its increased sensitivity to memory decline in MCI and AD populations (Crane et al., 2012). We performed mediation models to test whether NDI of the hippocampal cingulum mediated the relationship between AD pathology and the ADNI-MEM cognitive outcome measure. Our results showed that NDI of the hippocampal cingulum partially mediated the relationship between entorhinal tau and ADNI-MEM (indirect effect: β = -0.09, p = 0.036; direct effect: β = -0.64, p < 0.00001; total effect: β = -0.725, p < 0.00001; Fig. 3A). We also found that NDI of the hippocampal cingulum partially mediated relationships with ADNI-MEM in models of both meta temporal tau (indirect effect: β = -0.09, p = 0.024; direct effect: β = -0.59, p < 0.00001; total: β = -0.69, p < 0.00001; Fig. 3B) and Aβ ( indirect effect: β = -0.002, p = 0.005; direct effect: β = -0.006, p = 0.0005; total effect: β = -0.008, p < 0.00001; Fig. 3C). These mediation results further support the sensitivity of NDI to detect mechanistic relationships in AD. Additionally, we did not find significant associations between FA and either the predictor variables (tau or amyloid) or the outcome measures (cognition), which initially precluded construction of mediation models for FA. However, to fully address the possibility of indirect-only effects, we conducted exploratory mediation analyses using FA as a mediator. These models yielded nonsignificant indirect effects across all mediation pathways (entorhinal tau: p = 0.35; meta-temporal tau: p = 0.57; amyloid: p = 0.42), further supporting the absence of meaningful mediation for FA.

### 3.4 Combination of NODDI and tensor metrics best estimate clinical/cognitive outcomes

Our final goal was to test which combination of diffusion metrics best estimated cognitive outcome measures associated with AD, and to determine the strength of estimation of these models, using ROC analyses. First, we constructed separate models to compare NODDI and tensor metrics directly. Second, we took the top metric from each model to create a combined diffusion model to maximize estimations. Due to the significant association between outcomes such as memory and demographic variables, final combined models included participants’ age, sex, and education. Additionally, the separate metrics models were performed both with and without demographics included. On the models with demographics, there was only a slight change in the ROC (~0.03 difference), showing that inclusion of demographics in the independent metric model runs was not impacting the model in a positive nor negative way. Relative feature importance was also checked and demographics consistently scored at the middle to bottom of the table further showing the lack of impact of demographics on the individual models metrics selection.

**Fig. 4.**
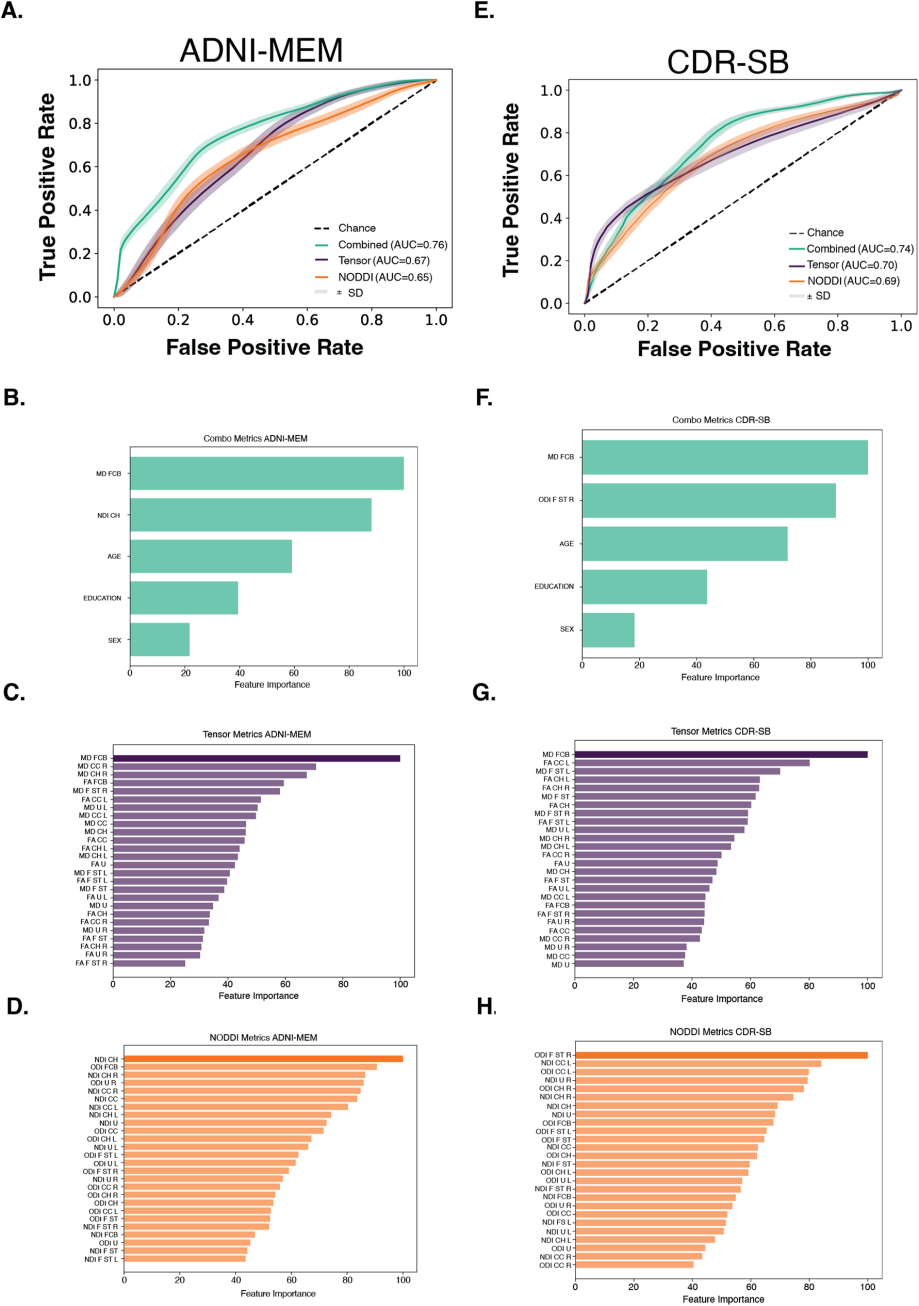
Characteristic curves for random forest classification models. (A) ADNI-MEM model showing combined metrics (green), tensor metrics (purple), and NODDI metrics (orange). (B) Gini importance table showing combined model results for ADNI-MEM. (C) Gini importance table showing just tensor metric results for ADNI-MEM. (D) Gini importance table showing only NODDI metrics results for ADNI-MEM. (E) CDR-SB model showing combined metrics (green), tensor metrics (purple), and NODDI metrics (orange). (F) Gini importance table showing combined model results for CDR-SB. (G) Gini importance table showing only tensor metric results for CDR-SB. (H) Gini importance table showing only NODDI metrics results for CDR-SB. Dotted line = chance. Combination models included age, gender, and education. Tensor and NODDI Gini Tables have top importance features bolded to show it was used in the combination modeling. Abbreviations: CC = cingulum cortex, CH = hippocampal cingulum, FCB = fornix column body, F ST = fornix stria terminalis, U = uncinate, R = right hemisphere, L=left hemisphere.

We first tested the estimation of memory performance using ADNI-MEM (Fig. 4A). Participants were divided into two subgroups using a median split method. Initial models were conducted separately for the NODDI metrics and tensor metrics, revealing distinct outcomes. The NODDI model (AUC = 0.65; CI 95% bootstrap: [0.649,0.651]) displayed a more even distribution of Gini importance values across all included tracts and metrics (Fig. 4D), whereas the tensor model (AUC = 0.67 [0.669,0.671]) had a clear top performer, with a 29% performance gap between MD cingulum cortex (71%) and MD fornix column body (100), the two highest ranking metrics on the importance table (Fig. 4C). For the combined model, NDI hippocampal cingulum and MD fornix column body were selected along with the demographic variables. This combined model resulted in the strongest estimation, with an AUC of 0.76 [0.759, 0.761].

We next tested estimation of the CDR-SB score (Fig. 4E). We first classified participants as having a score indicating impairment (CDR-SB > 0; n = 77) or no impairment (CDR-SB = 0; n = 115). The NODDI model had an AUC of 0.69 [0.689,0.691]. Within this model, ODI of the fornix ST had the highest Gini value and was, therefore, the best metric for estimating the outcome, compared with the other NODDI tract metrics (Fig. 4 H). The tensor model had an AUC of 0.70 [0.699,0.701]. MD of the fornix column body had the highest Gini importance index (Fig. 4G). The combined model, incorporating these top two performers, demonstrated an even higher estimation value for CDR-SB classification, yielding an AUC of 0.74 [0.739, 0.741]. Specificity and sensitivity for each model are given in Supplementary Table 3. CDR-MEM was not used as a model due to a lack of response diversity, making it an insufficient outcome measure to be used with a machine learning model.

Formal pairwise comparisons of the AUC curves were conducted using the roc.test function in R. For ADNI-Mem, the combined DTI+NODDI model demonstrated significantly higher AUC than the NODDI model alone (p = 0.01), and showed a trend toward outperforming DTI alone (p = 0.06). No significant AUC differences were observed between NODDI and DTI directly. For CDR-SB, no significant AUC differences were detected between any of the models.

## 4 Discussion

The utilization of multishell diffusion imaging has opened new doors to understanding AD in ways that were previously unattainable through standard diffusion tensor modeling techniques. Previous studies using standard single shell tensor models have left significant gaps in our understanding of the complexities of how WM microstructure is impacted by AD. Our research clearly highlights the advantages of employing the NODDI multishell compartmental model for gaining deeper insights into the pathology and cognitive outcomes of aging and AD. Our study demonstrates significant associations between NODDI and overall cognitive/clinical status, memory-related processes, and Alzheimer’s pathology. Overall, our study suggests that while single-shell DTI is a useful tool for studying aging, NODDI is able to give us additional information when it comes to cognition and pathology where tensor metrics such as FA fail to show significance. Exploratory subgroup analyses suggest that the observed associations are not solely driven by cognitively impaired individuals; however, smaller sample sizes within these subgroups may limit statistical power.

Our study delved into the association between NODDI and tensor metrics with outcomes specific to aging and AD. The NODDI measure NDI, specifically when measured in the hippocampal cingulum, demonstrated more significant associations with cognition and pathology than tensor measures. In contrast, FA did not exhibit many associations with cognition and pathology. NDI had strong associations specifically in the cingulum cortex and hippocampal cingulum regions for tau and Aβ burden. Mediation models demonstrated that NDI was a significant mediator of the relationship between ADNI-MEM and entorhinal tau, meta temporal tau, and β-amyloid, further supporting our hypothesis that the utilization of NODDI can show more specific results than those of just DTI alone as FA was unable to be used for mediation models due to lack of significant associations. Our results highlight significant associations between these NODDI metrics and tau pathology, with the NDI showing heightened sensitivity of entorhinal tau. Additionally, our results showed that Aβ had associations with the integrity of the cingulum, uncinate, and fornix across both NDI and ODI metrics.

Although DTI has been widely employed to describe WM properties in previous research, it is not without limitations, such as issues related to crossing fibers (Chen et al., 2023). It is widely recognized that as humans age, irrespective of the presence of neurodegenerative diseases, there is typically a reduction in fractional anisotropy (FA). However, FA can be a somewhat ambiguous metric, with the decline potentially attributable to various structural factors, including demyelination or a decrease in axon density (Sampaio-Baptista & Johansen-Berg, 2017). While the development of alternative diffusion analysis pipelines has helped mitigate some of these limitations associated with DTI, the application of NODDI has emerged as a valuable method for assessing microstructural changes (Chen et al., 2023). Our study demonstrates that FA alone exhibited weak, non-significant strength of association with nearly all of the study’s outcome measurements. Recently, a study conducted on the ADNI3 cohort by Chen et al. (2023) demonstrated that FA within specific MTL regions, particularly the hippocampal cingulum, exhibited correlations with tau burden. Our findings provide a more comprehensive examination of the compartmental complexity of WM, revealing that a stronger and more detailed association can be derived with NODDI than from the FA metric measurement alone. Based on this observation, we posit that NODDI metrics, with their capacity to discern alterations in microstructure, play a pivotal role in understanding the underlying changes occurring in WM during both the aging process and the progression of AD. While prior studies have demonstrated the utility of NODDI for detecting microstructural changes in Alzheimer’s disease and have compared NODDI with DTI in several contexts (Radhakrishnan et al., 2022), our findings add to this growing literature by systematically evaluating both tensor and NODDI models within a harmonized multisite cohort and testing mechanistic mediation models linking white matter changes with both pathology and cognition.

NDI may have increased sensitivity to detect early pathological changes compared with other metrics due to its ability to provide information at the cellular level. This gives information, which is achieved through NODDI’s use of multicompartment analysis via the acquisition of multishell imaging, at an anatomical level. This in turn is more nuanced and in depth when compared with the DTI methods which use a single diffusion shell to make more generalized statements about the status of water molecule movement within a region of tissue. Our study expands on previously published studies that investigated aging using NODDI and provides a more comprehensive assessment of how tensor metrics compare with multiple compartment approaches such as NODDI. One recent study found that both NODDI and tensor metrics are sensitive to age-related changes in WM regions that are associated with aging (Motovylyak et al., 2022). While our work is similarly focused on the differences between these two diffusion analyses methods, our goal was to identify which measures were more associated with Alzheimer’s pathology (measured by PET) and related cognitive outcomes, and not the aging process per se. Another recent study investigated the relationship between flortaucipir PET and WM health focusing on NDI as their primary metric (Tian et al., 2023). The authors showed that as Braak stages progressed, implying worsening levels of tau depositing, NDI in WM tracts decreased. Our findings build upon this previous work by demonstrating that utilizing a large multisite cohort study such as ADNI3 can allow for the extension of the previous work by comparing both cognitive and PET measurements within one study, with implications for follow-up studies to show longitudinality. Additionally, while our study focused primarily on the white matter changes, other studies such as Vogt et al. (2020) found a drop in NDI within the MCI group, further showing NODDIs utility for identifying cortical microstructural degeneration. Future studies could potentially use the ADNI3 cohort to further investigate those findings. We also recognize that the use of mediation models specifically with NODDI metrics is complicated and not to be easily reduced to a confirmatory metric. There are clearly multiple processes that may impact the physiological properties of the AD-related pathways we investigated, including axonal degeneration, demyelination, Wallerian degeneration secondary to gray matter neuronal loss, neuroinflammatory activity, vascular dysfunction, and extracellular matrix alterations. While our diffusion metrics are sensitive to microstructural change, they cannot fully disentangle the relative contributions of these mechanisms. Our work demonstrating the significance of NODDI in these models confirms this diffusion metric’s ability to detect alterations but cannot inform as to the specific cell types or structures involved. The present findings indicate that neither NDI nor ODI uniformly outperformed the other across all medial temporal tracts. Rather, the two metrics demonstrated region-specific associations, supporting the utility of examining both parameters when characterizing white matter microstructural changes in Alzheimer’s disease.

Although previous studies have examined associations between NODDI metrics and Alzheimer’s pathology, our work extends these findings by simultaneously evaluating both tensor and NODDI-derived diffusion metrics across multiple medial temporal white matter pathways using a harmonized, multisite ADNI3 sample. In addition to characterizing region-specific associations, we employed mediation analyses to explore mechanistic relationships linking white matter microstructure with amyloid and tau pathology as well as cognitive outcomes. Furthermore, our integration of machine learning approaches provides an initial assessment of the predictive utility of these diffusion metrics, offering new insights for potential future clinical applications.

Finally, we tested the utility of NODDI and tensor metrics in estimating cognitive outcomes. We used the general rule that an AUC of 0.5 suggests no ability to estimate, and 0.7 to 0.8 is acceptable estimation (Sampaio-Baptista & Johansen-Berg, 2017). NODDI metrics, notably the NDI of the hippocampal cingulum, proved to be superior estimations of clinical outcomes when compared with demographic variables such as age, gender, and education. Similarly, tensor metrics, specifically the MD of the right fornix, exhibited acceptable estimative performance. The fusion of these metrics with demographic information yielded increased estimation models characterized with higher AUC values, suggesting models combining both NODDI and tensor metrics have increased sensitivity in the estimation of cognitive performance. These results suggest that while NODDI and DTI individually perform comparably for classifying cognitive performance, the combination of both diffusion models provides modest but significant incremental improvement.

Currently, within the clinical trial and diagnostic realms, structural MRI serves as a crucial step in the process of confirming AD staging (Jack et al., 2010). However, while volumetric measurements are employed to identify gray matter atrophy, the characterization of WM integrity can add more information about how neural communication and networks may be impacted. Our study underscores the value of integration of multishell diffusion acquisitions into the ADNI3 protocol, which has enabled a more complete understanding of the neurodegenerative processes occurring in both the aging population and individuals with AD. These metrics also open up new avenues for future clinical trials, offering the potential to quantify early disease markers that were previously beyond reach using standard dMRI sequences. This advance holds promise for enhancing our ability to detect and address neurodegenerative conditions at earlier stages, potentially improving therapeutic interventions (Jack et al., 2010).

The study had several limitations that are important to note. First, the ADNI3 sample is not particularly diverse with respect to race and ethnicity. While our cohort includes individuals across a range of cognitive status, including mild dementia, this design was intentional to permit modeling of continuous relationships across the full AD spectrum, rather than restricting to isolated preclinical or prodromal stages. Our machine learning models would greatly benefit from a broader and more diverse dataset, as machine learning is not expected to generalize well to other samples if the training dataset is not sufficiently diverse (Gong et al., 2018). Second, due to the sensitivity of neuroimaging to movement and imaging artifacts, our sample size was limited due to the need to censor participant data that did not pass quality control. Additionally, while we limited data to Siemens Prisma scanners to minimize inter-scanner variability, we formally tested for residual site differences in NODDI measures. These analyses did not identify significant site effects, supporting the comparability of diffusion metrics across sites. The decision to use only a single shell during the DTI analysis was because prior ADNI cohorts only collected a single b = 1000 shell. One goal of this paper is to stress the importance of continuing to include multishell sequences such as the newly added ADNI3 advanced protocol. We would like to recognize that utilizing multishell or even a higher b-shell for the DTI analysis would more than likely improve the strength of the DTI results. While the fornix ST remains susceptible to partial volume effects due to its small size, we applied visual quality control and used NODDI modeling to mitigate these concerns by accounting for tissue compartmentalization within each voxel; however, some residual contamination cannot be fully excluded. Further, while our study focused on WM within the MTL, future work should extend these analyses to whole-brain tractography to assess whether the observed sensitivity of NODDI metrics in MTL tracts generalizes across other white matter pathways. For this study, we chose to only focus on four DWI analysis metrics (FA, MD, NDI, and ODI). Future studies should branch out to include additional metrics for both NODDI and DTI analyses (such as free-water isotropic volume fraction, radial diffusivity, axial diffusivity) as well as potentially investigating other analyses such as Diffusion Kurtosis Imaging (DKI). Finally, neuroimaging studies investigating early stages of neurocognitive change with aging are limited by the relatively low sensitivity of neuropsychological and clinical measures of impairment. The use of more sensitive cognitive tasks is warranted in future studies.

In conclusion, our study offers new insights into the estimation potential of NODDI metrics in studies of AD. While tensor metrics have traditionally served as the standard for leveraging dMRI in assessing alterations in pathology and memory related to AD, our research suggests that conventional FA and MD measurements may miss more subtle microstructural changes. Notably, NDI emerges as an exceptionally sensitive metric, particularly in the context of entorhinal tau pathology and memory-related outcome measures. This underscores the potential of NODDI metrics to uncover subtle yet crucial insights into the complex dynamics of neurodegenerative processes. Taken together, these findings suggest that multimodal diffusion models enhance classification performance, whereas NODDI measures in particular provide more sensitive and mechanistically interpretable markers of MTL white matter integrity in AD. Thus, these approaches should be considered complementary, with combined models maximizing predictive accuracy and NODDI contributing additional biological specificity.

## Supplementary Material

Supplementary Material

## Data Availability

All imaging and outcome measures are available publicly via Alzheimer’s Disease Neuroimaging Initiative. For up-to-date information and the most recent datasets, see: www.adni-info.org.
