# Supplementary material for: NODDI-derived measures of microstructural integrity in medial temporal lobe white matter pathways are associated with Alzheimer’s disease pathology and cognition
Source: Imaging Neurosci (Camb). 2025 Oct 23;3:IMAG.a.950. doi: 10.1162/IMAG.a.950 (PMC12550277; doi:10.1162/IMAG.a.950)

Supplemental Tables

Supplementary Figure 1A: NODDI Metrics Partial Correlations with raw p-values

	ADNI-MEM	CDRSB	CDR-MEM	Aβ	Entorhinal	Meta Temporal	Hippocampal Volume
**NDI CC**	0.233 (0.0017)	-0.308 (3.05E-05)	-0.291 (3.69E-05)	-0.239 (0.0067)	-0.266 (0.0033)	-0.168(0.065)	-0.137 (0.0975)
**NDI CH**	0.343(8.52E-06)	-0.367 (6.66E-07)	-0.325 (3.62E-06)	-0.332 (0.00023)	-0.375(2.32E-05)	-0.254 (0.015)	-0.226 (0.0125)
**NDI Fornix Col Body**	0.126 (0.1075)	-0.263(0.000342)	-0.240 (0.00072)	-0.153(0.08375)	-0.207 (0.02)	-0.228 (0.02)	0.092 (0.197)
**NDI Fornix ST**	0.106 (.15)	-0.093(0.195)	-0.101(0.162)	-0.068(0.417)	-0.192 (0.026)	-0.152(0.080)	-0.215 (0.0125)
**NDI Uncinate**	0.250 (0.00145)	0.250 (0.000523)	-0.234 (0.001)	-0.277 (0.00190)	-0.373(2.32E-05)	-0.199 (0.035)	-0.142 (0.0975)
**ODI CC**	0.075 (0.308)	0.031(0.663)	0.015(0.836)	-0.041(0.625)	-0.063(0.47)	-0.160 (0.1067)	-0.112 (0.24833)
**ODI CH**	0.164 (0.03125)	-0.179 (0.015)	-0.256 (0.028468)	-0.073(0.48)	-0.147(0.11375)	-0.132 (0.128)	-0.073 (0.44)
**ODI Fornix Col Body**	0.228 (0.005)	-0.216 (0.0033)	-0.207 (0.004)	-0.223 (0.0175)	-0.190 (0.0633)	-0.133 (0.128)	0.190 (0.0175)
**ODI Fornix ST**	0.253 (0.00247)	-0.253 (0.00091)	-0.256 (0.00031)	-0.252 (0.01118)	-0.288 (0.004)	-0.319 (0.00085)	-0.301(2.00E-06)
**ODI Uncinate**	0.218 (0.005)	-0.255 (0.0009)	-0.195 (0.006)	-0.151(0.070)	-0.179 (0.06333)	-0.162 (0.10667)	-0.027(0.734)

R-value (P value); hippocampal volume was corrected with individual participants' intracranial volume. All p-values corrected with FDR.

Supplementary Table 2A. Tensor metrics partial correlations

	ADNI-MEM	CDRSB	CDR-MEM	Aβ	Entorhinal	Meta	Hippocampal Volume
**FA CC**	0.057 (0.62875)	-0.160 (0.0625)	-0.136 (0.058)	-0.076 (0.455)	-0.069 (0.429)	0.047 (0.7388)	0.009 (0.911)
**FA CH**	0.049 (0.62875)	-0.061 (0.396)	-0.042 (0.563)	-0.171 (0.1)	-0.082 (0.429)	-0.024 (0.784)	-0.086 (0.3375)
**FA Fornix Col Body**	0.232 (0.005)	-0.198 (0.025)	-0.240 (0.000729)	-0.190 (0.1)	-0.181 (0.18)	-0.211 (0.075)	0.552 (1.43E-16)
**FA Fornix ST**	0.084 (0.62875)	-0.61 (0.396)	-0.077 (0.285)	-0.002 (0.98)	-0.126 (0.2733)	-0.129 (0.345)	-0.249 (0.0025)
**FA Uncinate**	-0.032 (0.662)	0.062 (0.396)	0.020 (0.776)	-0.111 (0.305)	-0.121 (0.2733)	-0.052 (0.739)	-0.169 (0.04833)
**MD CC**	-0.138 (0.075)	0.137 (0.07)	0.117 (0.103)	0.114 (0.173)	0.153 (0.077)	0.096 (0.325)	0.064 (0.412)
**MD CH**	-0.185 (0.02)	0.211 (0.005)	0.159 (0.026)	0.288(0.0011622)	0.167 (0.06625)	0.086 (0.325)	0.143 (0.0825)
**MD Fornix Col Body**	-0.311 (7.61E-05)	0.255 (0.0015797)	0.306 (1.35E-05)	0.287 (0.0011622)	0.197 (0.0367)	0.202 (0.0475)	-0.629(1.34E-22)
**MD Fornix ST**	-0.273 (0.00041)	0.234 (0.0025)	0.247 (0.000505)	0.174 (0.04625)	0.290 (0.0033)	0.349 (0.00018)	0.452 (9.44E-17)
**MD Uncinate**	-0.108 (0.142)	0.109 (0.131)	0.112 (0.120)	0.244 (0.005)	0.248 (0.01)	0.169 (0.085)	0.248 (0.0017)

R-value (P value); hippocampal volume was corrected with individual participants' intracranial volume. All p-values corrected with FDR.

Table 3. Model performance evaluation metrics

Classification	Features	AUC [CI]	Sensitivity (%) [CI]	Specificity (%) [CI]
ADNI-MEM	NODDI	0.65 [0.649 to 0.651]	62 [0.619 to 0.621]	65 [0.619 to 0.621]
	Tensor	0.67 [0.669 to 0.671]	59 [0.589 to 0.591]	65 [0.649 to 0.651]
	Combination	0.76 [0.759 to 0.761]	69 [0.689 to 0.691]	71 [0.709 to 0.711]
CDR-SB				
	NODDI	0.69 [0.689 to 0.691]	60 [0.599 to 0.601]	69 [0.689 to 0.691]
	Tensor	0.70 [0.699 to 0.701]	68 [0.678 to 0.682	70 [0.699 to 0.701]
	Combination	0.74 [0.739 to 0.741]	63 [0.629 to 0.631]	70 [0.699 to 0.701]

AUC = Area under the curve, CI = 95% Confidence Intervals, ADNI-MEM = Alzheimer's Disease Neuroimaging Initiative Memory composite score, CDR-SB = Clinical Dementia Rating Scale Sum of Boxes

Supplementary Table 4A. NODDI exploratory subgroup analyses of cognitively unimpaired

	ADNI-MEM	CDRSB	CDR-MEM	Aβ	Entorhinal	Meta Temporal	Hippocampal Volume
**NDI CC**	-0.111(0.24)	0.183(0.04)	0.073(0.43)	-0.26(0.01)	-0.188(0.09)	-0.188(0.09)	-0.045(0.6)
**NDI CH**	-0.009(0.93)	0.127(0.17)	0.04(0.67)	-0.24(0.02)	-0.127(0.2)	-0.13(0.26)	0.003(0.9)
**NDI Fornix Col Body**	-0.04(0.69)	0.018(0.85)	-0.09(0.33)	-0.19(0.06)	0.22(0.05)	-0.22(0.05)	0.076(0.4)
**NDI Fornix ST**	0.034(0.72)	0.03(0.75)	-0.06(0.51)	0.01(0.9)	0.008(0.9)	0.008(0.94)	0.07(0.5)
**NDI Uncinate**	-0.000007(0.99)	0.01(0.91)	-0.026(0.77)	-0.156(0.14)	-0.09(0.4)	-0.09(0.4)	-0.06(0.5)
**ODI CC**	0.191(0.04)	-0.05(0.61)	-0.028(0.76)	-0.05(0.63)	0.05(0.67)	0.05(0.7)	0.117(0.2)
**ODI CH**	-0.02(0.81)	0.03(0.69)	0.09(0.33)	0.06(0.55)	-0.04(0.7)	-0.04(0.7)	0.157(0.09)
**ODI Fornix Col Body**	0.04(0.70)	0.04(0.61)	0.06(0.52)	-0.09(0.42)	-0.07(0.5)	-0.07(0.5)	0.102(0.3)
**ODI Fornix ST**	-0.03(0.76)	0.03(0.75)	0.02(0.79)	-0.12(0.24)	-0.02(0.9)	-0.02(0.86)	0.235(0.01)
**ODI Uncinate**	-0.01(0.88)	0.175(0.06)	0.20(0.03)	-0.02(0.82)	-0.02(0.9)	-0.02(0.9)	0.012(0.9)

R-value (P value); hippocampal volume was corrected with individual participants' intracranial volume.

Supplementary Table 4B. NODDI exploratory subgroup analyses of cognitively impaired

	ADNI-MEM	CDRSB	CDR-MEM	Aβ	Entorhinal	Meta Temporal	Hippocampal Volume
**NDI CC**	0.23(0.06)	-0.261(0.03)	-0.1678(0.2)	0.06(0.66)	-0.136(0.3)	-0.066(0.6)	-0.024(0.8)
**NDI CH**	0.48(0.00003)	-0.388(0.00070)	-0.256(0.03)	-0.13(0.37)	-0.38(0.006)	-0.272(0.05)	0.318(0.005)
**NDI Fornix Col Body**	0.133(0.3)	-0.315(0.007)	-0.249(0.03)	0.019(0.9)	-0.182(0.2)	-0.26(0.06)	0.003(0.9)
**NDI Fornix ST**	0.006(0.9)	-0.06(0.6)	-0.038(0.8)	0.104(0.5)	-0.203(0.1)	-0.175(0.21)	0.05(0.7)
**NDI Uncinate**	0.34(0.004)	-0.278(0.01)	-0.189(0.1)	-0.059(0.7)	-0.411(0.002)	-0.214(0.1)	0.207(0.07)
**ODI CC**	-0.005(0.97)	0.04(0.7)	-0.015(0.9)	-0.026(0.9)	-0.144(0.3)	-0.252(0.07)	0.074(0.5)
**ODI CH**	0.28(0.02)	-0.186(0.1)	-0.092(0.4)	-0.115(0.43)	-0.153(0.3)	-0.11(0.4)	-0.049(0.7)
**ODI Fornix Col Body**	0.25(0.04)	-0.182(0.1)	-0.1207(0.3)	-0.17(0.2)	-0.195(0.2)	-0.111(0.4)	0.033(0.8)
**ODI Fornix ST**	0.37(0.002)	-0.202(0.09)	-0.143(0.2)	-0.10(0.5)	-0.421(0.002)	-0.469(0.0005)	0.254(0.02)
**ODI Uncinate**	0.363(0.002)	-0.343(0.003)	-0.229(0.05)	-0.117(0.4)	-0.245(0.08)	-0.229(0.102)	0.223(0.05)

R-value (P value); hippocampal volume was corrected with individual participants' intracranial volume.

Supplementary Table 5A. DTI exploratory subgroup analyses of cognitively unimpaired

	**ADNI-MEM**	**CDRSB**	**CDR-MEM**	**Aβ**	**Entorhinal**	**Meta**	**Hippocampal Volume**
**FA CC**	-0.168(0.07)	0.121(0.2)	0.061(0.509)	-0.048(0.6)	-0.096(0.4)	-0.008(0.9)	-0.097(0.3)
**FA CH**	0.012(0.9)	0.013(0.9)	-0.047(0.6)	-0.214(0.04)	-0.049(0.7)	0.069(0.5)	-0.171(0.06)
**FA Fornix Col Body**	0.124(0.2)	0.08(0.4)	-0.144(0.1)	-0.040(0.7)	-0.073(0.5)	-0.032(0.8)	0.329(0.00026)
**FA Fornix ST**	0.068(0.5)	-0.001(0.9)	-0.09(0.3)	0.046(0.7)	-0.022(0.8)	0.024(0.8)	0.059(0.5)
**FA Uncinate**	0.016(0.9)	-0.143(0.1)	-0.214(0.02)	-0.058(0.6)	-0.089(0.4)	-0.073(0.5)	0.0005(0.9)
**MD CC**	-0.019(0.8)	-0.122(0.2)	-0.054(0.6)	0.1303(0.2)	0.130(0.3)	0.1207(0.3)	0.1798(0.05)
**MD CH**	0.024(0.8)	-0.008(0.9)	0.027(0.8)	0.267(0.009)	0.126(0.3)	-0.021(0.9)	0.1615(0.08)
**MD Fornix Col Body**	-0.188(0.05)	-0.046(0.6)	0.148(0.1)	0.093(0.4)	0.079(0.5)	0.082(0.5)	-0.409(.00001)
**MD Fornix ST**	-0.121(0.2)	-0.027(0.8)	0.095(0.3)	0.018(0.9)	0.066(0.6)	0.136(0.2)	-0.292(0.0013)
**MD Uncinate**	-0.035(0.7)	0.1148(0.2)	0.207(0.03)	0.099(0.3)	0.216(0.06)	0.200(0.08)	-0.056(0.5)

R-value (P value); hippocampal volume was corrected with individual participants' intracranial volume.

Supplementary Table 5B. DTI exploratory subgroup analyses of cognitively impaired

	**ADNI-MEM**	**CDRSB**	**CDR-MEM**	**Aβ**	**Entorhinal**	**Meta**	**Hippocampal Volume**
**FA CC**	0.109(0.4)	-0.160(0.2)	-0.066(0.6)	0.0390(0.8)	0.0208(0.9)	0.1277(0.4)	-0.0372(0.8)
**FA CH**	0.148(0.2)	-0.173(0.1)	-0.136(0.2)	-0.0249(0.9)	-0.1389(0.3)	-0.1071(0.5)	0.2734(0.02)
**FA Fornix Col Body**	0.213(0.08)	-0.175(0.1)	-0.190(0.1)	-0.139(0.3)	-0.0645(0.6)	-0.1695(0.2)	0.5595(.00001)
**FA Fornix ST**	0.015(0.9)	-0.048(0.7)	-0.036(0.8)	0.1379(0.3)	-0.0723(0.6)	-0.1419(0.3)	0.4241(0.00013)
**FA Uncinate**	-0.14(0.2)	0.1397(0.2)	0.0851(0.5)	-0.0828(0.6)	-0.115(0.4)	-0.067(0.6)	0.1169(0.3)
**MD CC**	-0.134(0.3)	0.132(0.3)	0.059(0.6)	-0.0362(0.8)	0.0716(0.6)	0.0234(0.9)	0.0171(0.9)
**MD CH**	-0.434(0.0002)	0.3142(0.007)	0.1768(0.1)	0.1102(0.5)	0.1397(0.3)	0.0976(0.5)	-0.1460(0.2)
**MD Fornix Col Body**	-0.221(0.07)	0.1531(0.2)	0.158(0.1)	0.198(0.2)	0.0263(0.9)	0.0782(0.6)	-0.5557(.00001)
**MD Fornix ST**	-0.204(0.09)	0.1083(0.4)	0.0703(0.5)	-0.031(0.8)	0.1931(0.2)	0.3131(0.02)	-0.5012(.002)
**MD Uncinate**	-0.122(0.3)	0.1495(0.2)	0.1470(0.2)	0.2295(0.1)	0.2692(0.05)	0.2484(0.08)	-0.3565(0.002)

R-value (P value); hippocampal volume was corrected with individual participants' intracranial volume.

Supplementary Table 6A. NODDI Scatter Plots of Cognitive Outcome Measures

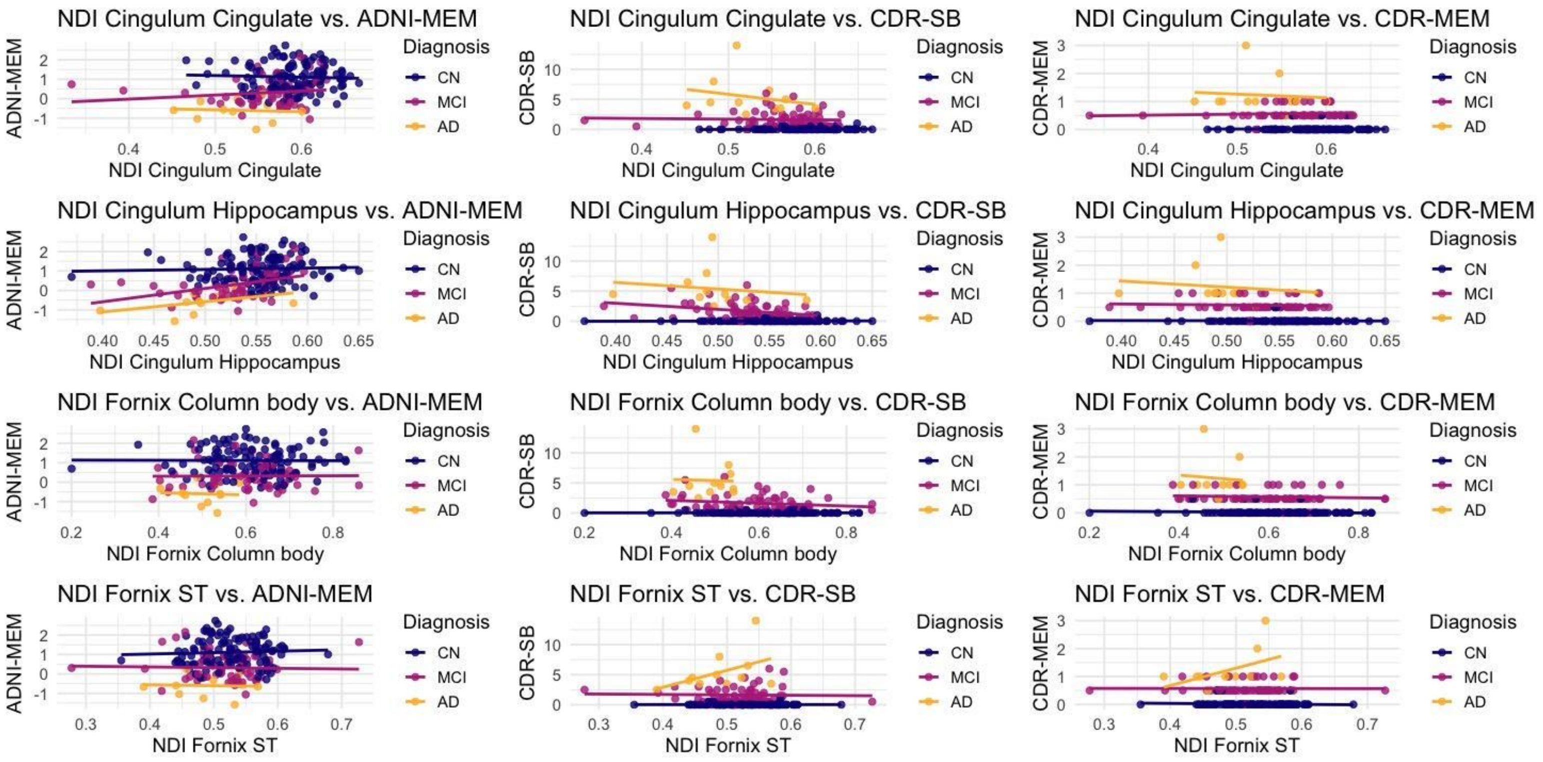

Supplementary Table 6B. ODI Scatter Plots of Cognitive Outcome Measures

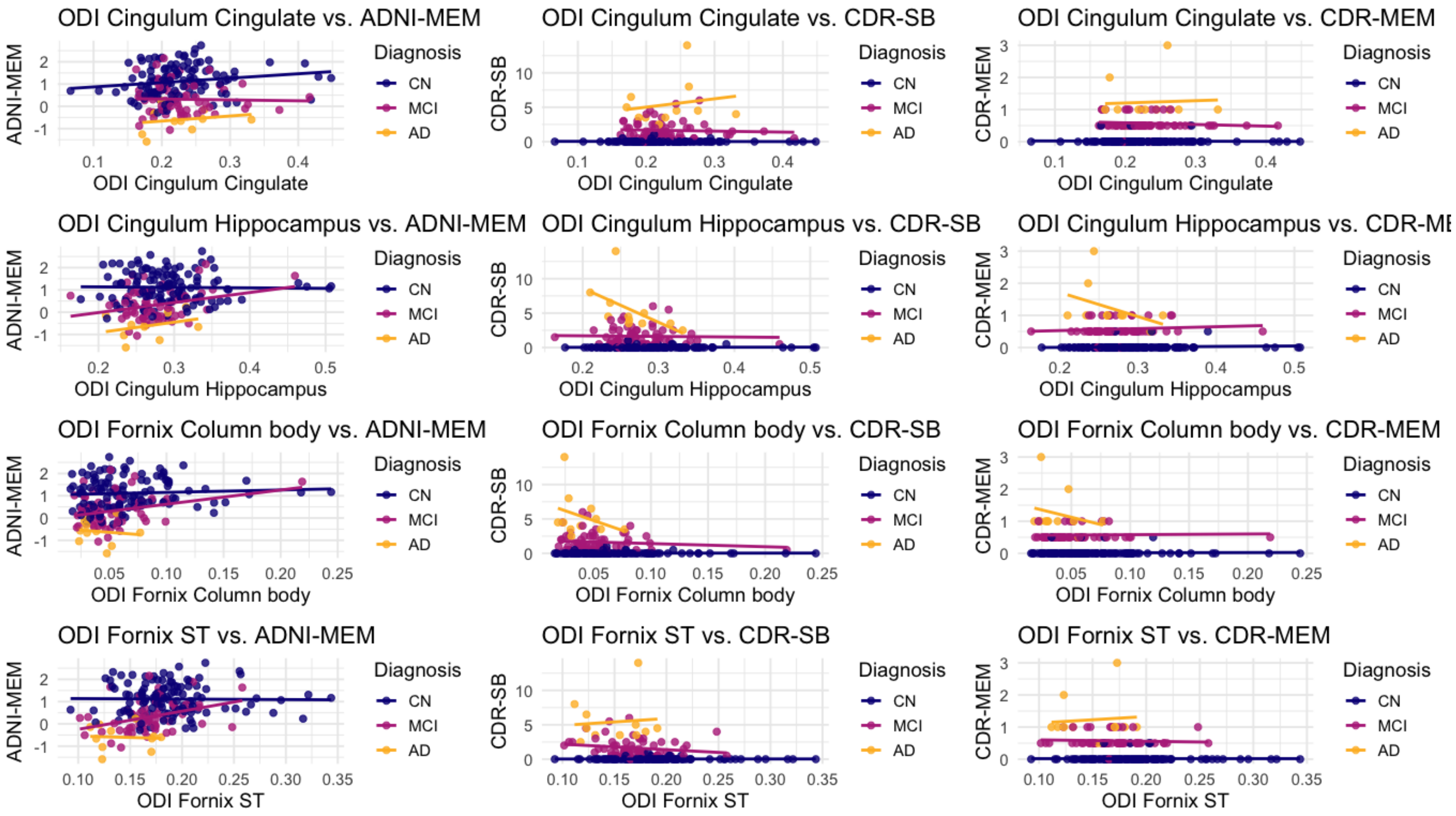

Supplementary Table 7A. FA Scatter Plots of Cognitive Outcome Measures

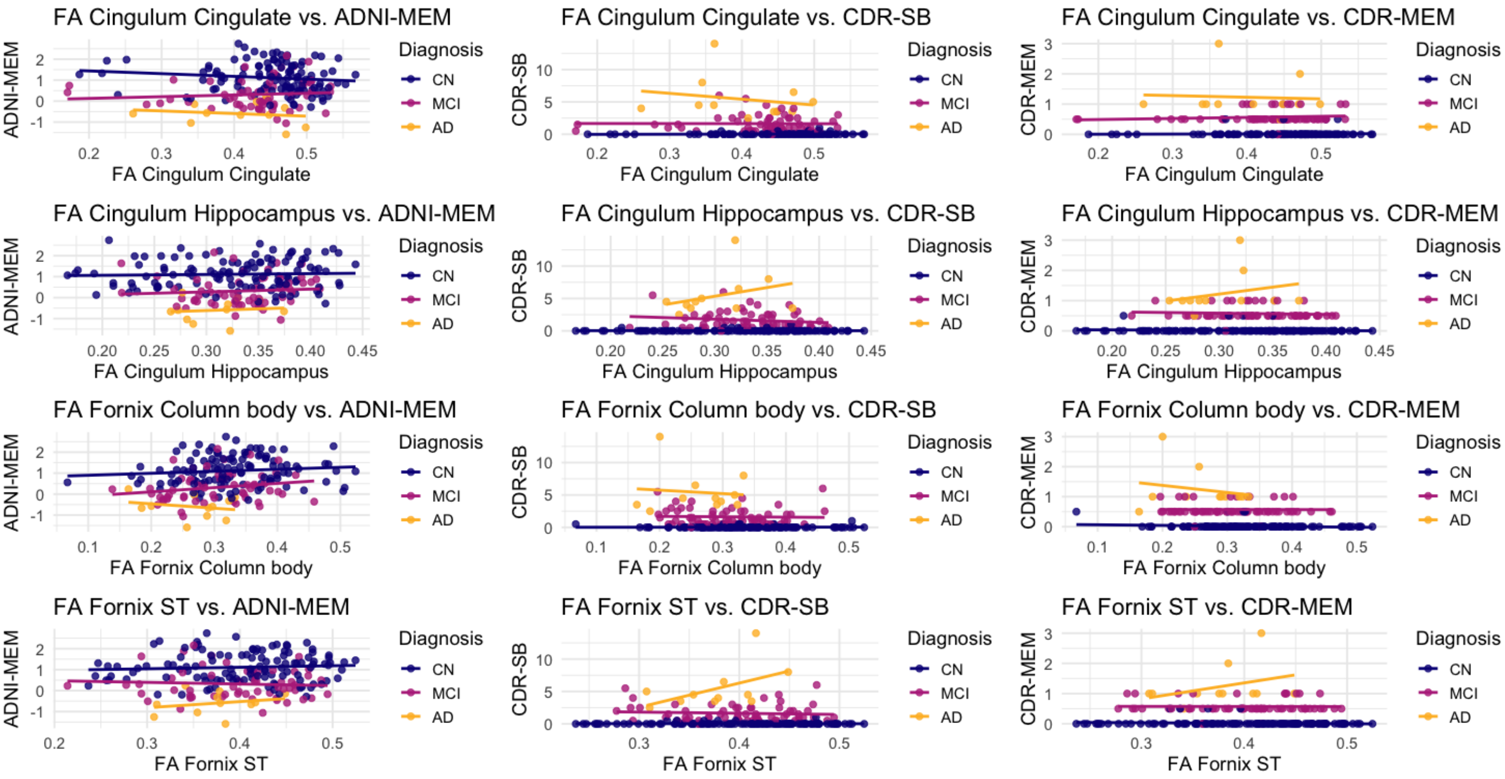

Supplementary Table 7B. MD Scatter Plots of Cognitive Outcome Measures

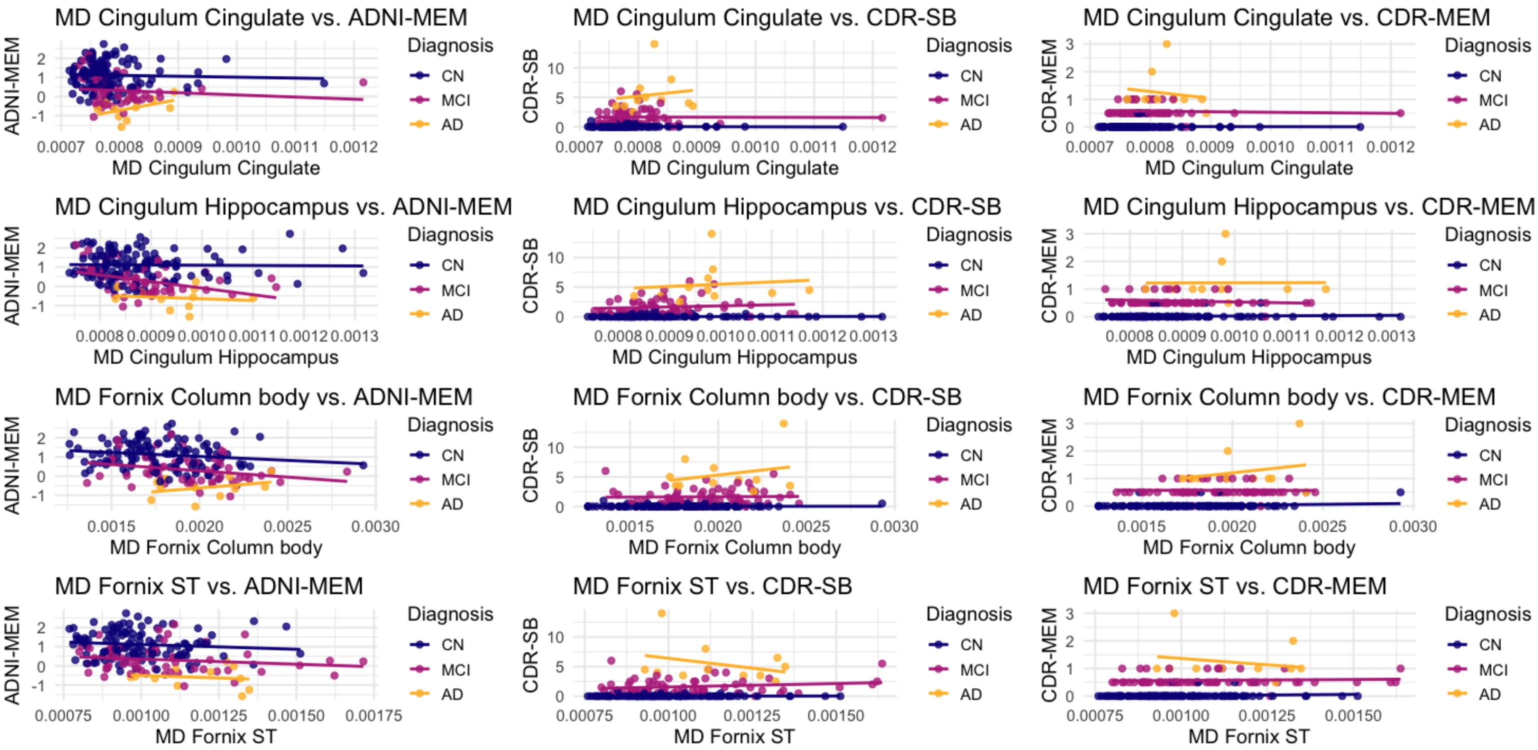

Supplementary Table 8A. NDI Scatter Plots of AD Biomarker

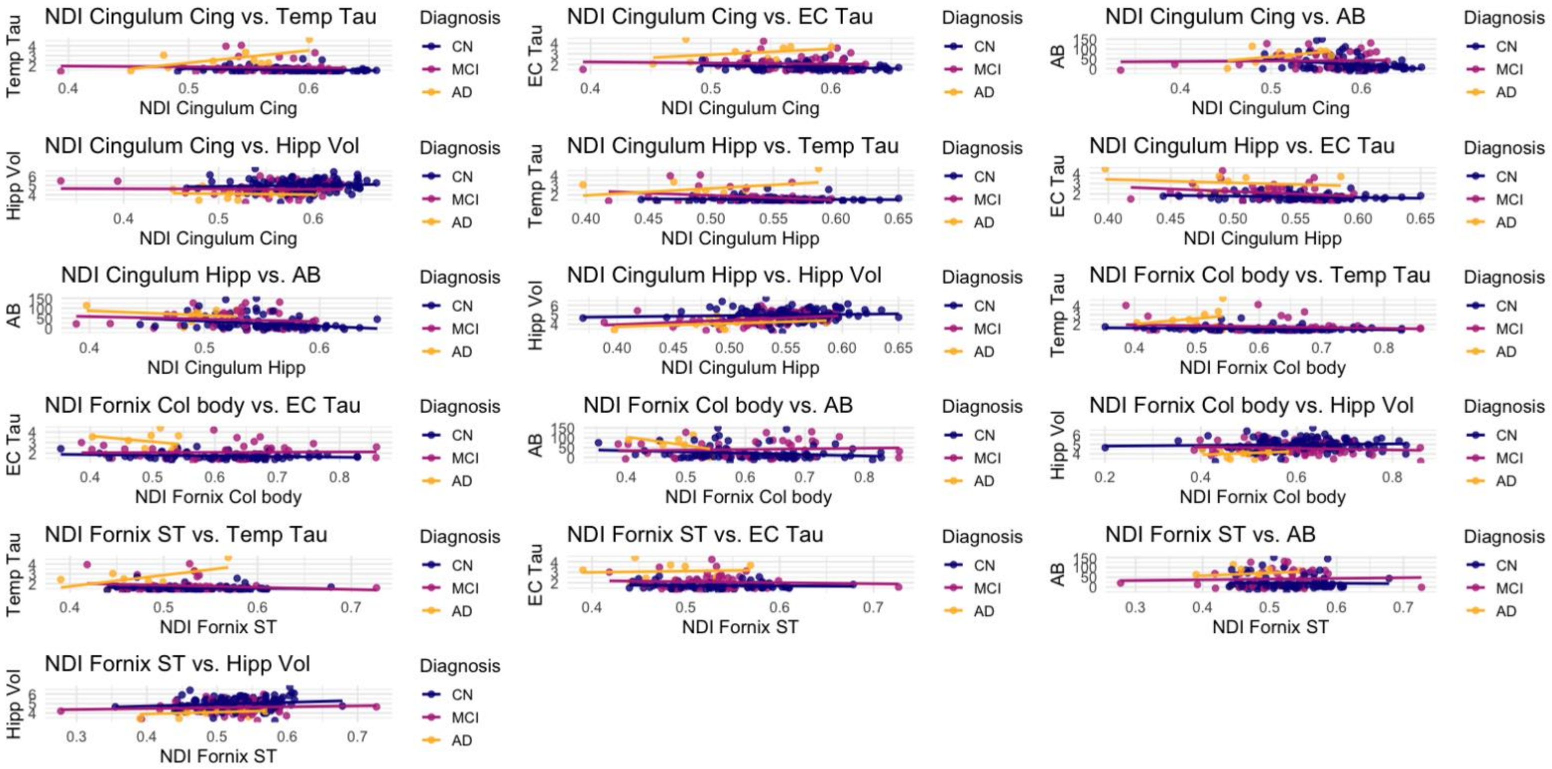

Supplementary Table 8B. ODI Scatter Plots of AD Biomarker

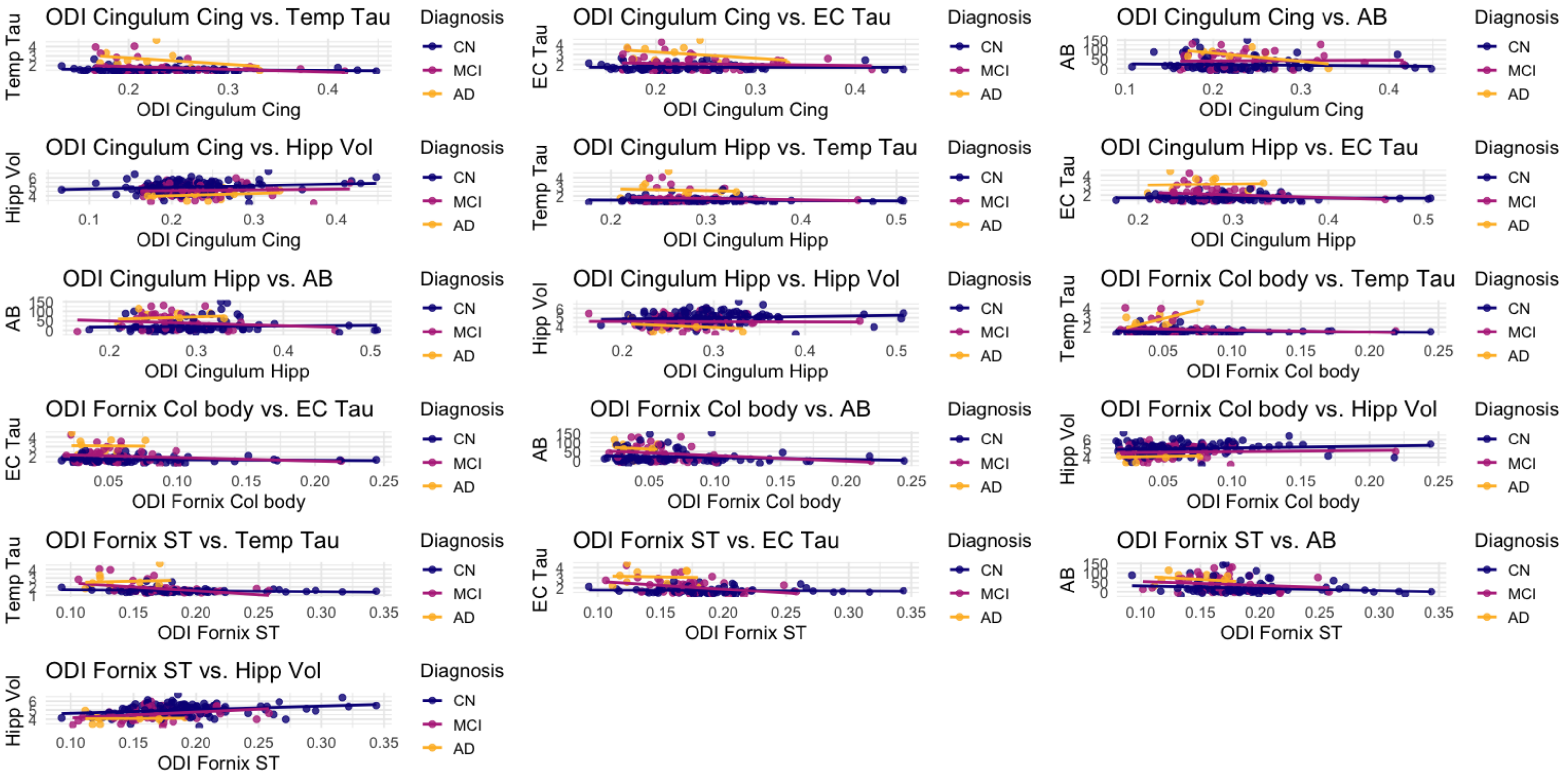

Supplementary Table 9A. FA Scatter Plots of AD Biomarkers

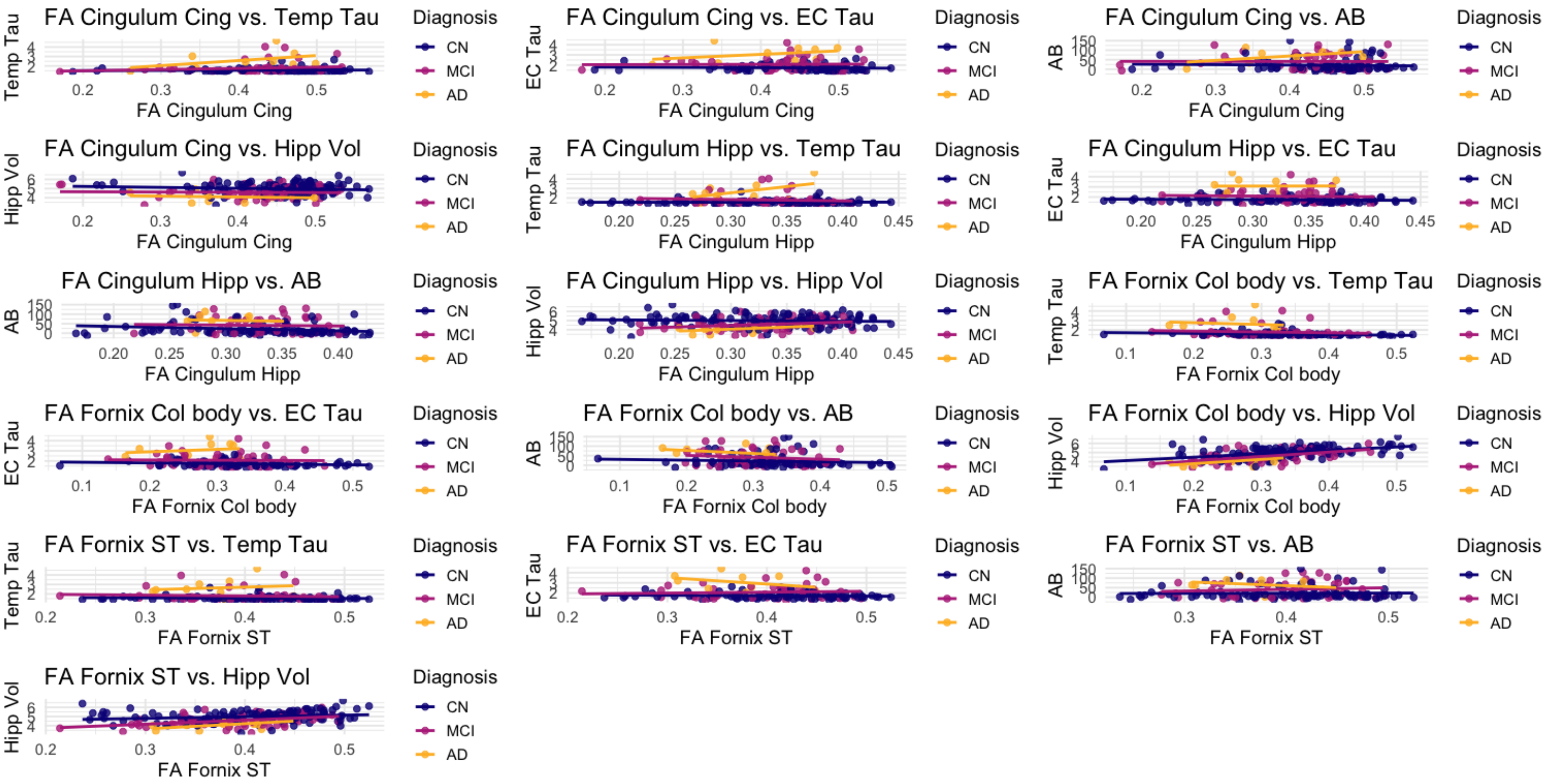

Supplementary Table 9B. MD Scatter Plots of AD Biomarkers

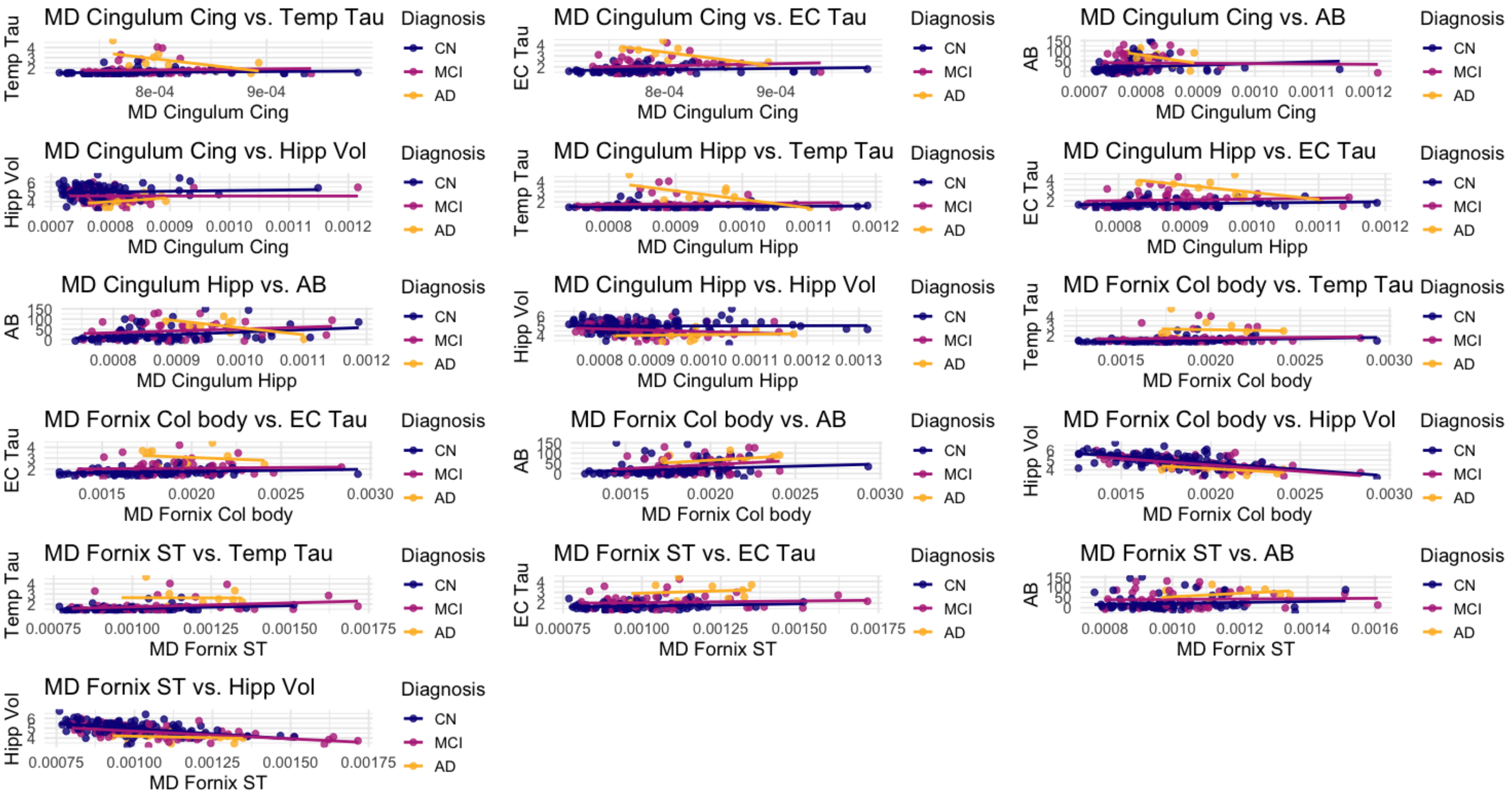

Supplement: Supplementary Material [file IMAG.a.950_supp.pdf]
